# Highlights of *Toxoplasma gondii* research papers published in *Parasitology* in the last 5 decades: personal perspective

**DOI:** 10.1017/S0031182025000186

**Published:** 2025-03

**Authors:** Jitender P. Dubey

**Affiliations:** U.S. Department of Agriculture, Agricultural Research Service, Animal Parasitic Diseases Laboratory, Beltsville Agricultural Research Center, Beltsville, MD, USA

**Keywords:** abortion, behaviour, congenital, chemotherapy, diagnosis, epidemiology, molecular, transmission, toxoplasma gondii, vaccine

## Abstract

**Figure:**
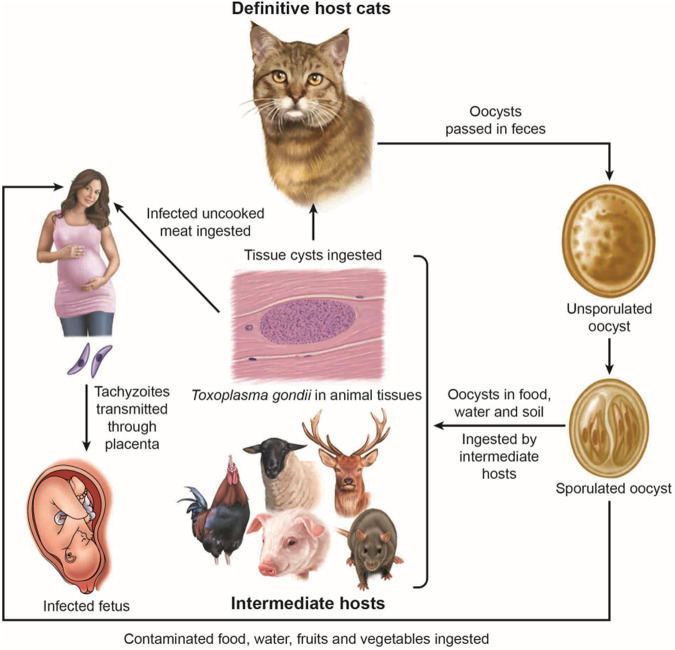

## Introduction

### General comment

I am honoured to write this editorial. I have published in *Parasitology* since 1976 on various parasite topics but mostly on *Toxoplasma gondii*. Before commenting on *T. gondii* papers, I would like to comment on the journal itself and why I published papers in *Parasitology*.

1. *Parasitology* is highly reputed and well serves the discipline of parasitology and health sciences in general. The journal was founded in 1908, and it coincides with the discovery of the parasite, *Toxoplasma* in the same year by Nicolle and Manceaux (1908). Professor Nuttal was its first editor; he had also founded *The Journal of Hygiene* in 1901.
2. The editors and the editorial staff were very helpful to young scientists, especially from nations whose mother tongue was not English (including me). During my 5 decades of association with the journal, many editors helped me. Additionally, the services of 2 editorial assistants standout: Dr Elizabeth Painter served the journal for 33 years, first as an editorial assistant and later as an assistant editor; she was meticulous, going through each paper line by line. I phoned her many times, seeking help and explanation. Alison Sage now admirably serves a similar function.
3. There was no limit to the length of a paper to be published in *Parasitology*; the very first article in the journal is more than 100 printed pages. Some of the papers I published are very long.
4. Until recently, there were no page charges, and the costs of publishing colour illustrations were often waived; I was a beneficiary of that.
5. The reviews of papers were meticulous but fair.

### Toxoplasma gondii *papers selected for review*

*Parasitology* published more than 250 papers on *T. gondii* in the 5 decades after discovery of its full life cycle, in 1970. I selected the following papers because of their importance and personal knowledge. I could not include other meritorious papers in the interests of brevity.

1. **Cornelissen AWCA, Overdulve JP, and Hoenderboom JM** (1981) Separation of *Isospora (Toxoplasma) gondii* cysts and cystozoites from mouse brain tissue by continuous density-gradient centrifugation. *Parasitology* **83**, 103–108, PMID: 6267543.

Most experiments with *T. gondii* had been performed using the tachyzoite stage because large numbers can be obtained in mice or in cell culture. Tissue cysts and contained bradyzoites are difficult to cultivate and not easy to purify. This paper determined the specific gravity (1.056) of *T. gondii* tissue cysts, using high-speed centrifugation gradients, and developed a method to separate intact tissue cysts from mouse brains using Percoll gradients. They showed that isolated tissue cysts were alive after Percoll separation and that the cysts from older infections were more easily separated from mouse brain than small cysts.

This method enables collection of almost pure bradyzoites after treating intact tissue cysts with acidic pepsin, and the technique has been applied to separation of *Sarcocystis* species bradyzoites (Dubey et al., 2016) and collection of *Hammondia hammondi* and *Besnoitia* species bradyzoites (J. P. Dubey, own observations).

2. **Couvreuer G, Sasak A, Fortier B, and Dubremetz JF** (1988) Surface antigens of *Toxoplasma gondii. Parasitology* **97**, 1–10, PMID: 3174230.

Discovering a novel serological test, the dye test by Sabin and Feldman (1948) opened the way for *Toxoplasma* research worldwide; its use revealed that *T. gondii* infections are prevalent in humans worldwide and are mostly asymptomatic. This test is highly specific and sensitive and was used as the Gold Standard for the discovery of other serological tests. However, the dye test requires use of live tachyzoites of a highly pathogenic strain, the RH strain. In 1980, studies were pioneered by Handman et al. (1980), Kasper et al. (1983) and Kasper (1987) who characterized 4 major surface antigens of *T. gondii* tachyzoites (P43, P35, P30 and P22); these results showed a great promise for the development of new diagnostic tests and prospects for vaccines. However, identification of these antigens was not always clear cut. This paper developed monoclonal antibodies against these peculiar surface antigens using hybridomas. These antigens were further characterized by immunofluorescence on living tachyzoites. Their results confirmed the existence of the 4 major antigens characterized previously and added another surface antigen, P23.

The simultaneous characterization of these 5 surface antigens provided a better definition for these antigens. Findings have been helpful in the development of new serological methods for diagnosis of toxoplasmosis.

3. **Webster JP, Brunton CF, and MacDonald DW** (1994) Effect of *Toxoplasma gondii* upon neophobic behaviour in wild brown rats, *Rattus norvegicus. Parasitology* **109**, 37–43, PMID: 8058367.

*Toxoplasma gondii* is one of the most successful parasites, being ubiquitous and infecting virtually all endotherms, including humans. Parts of this successful transmission entails transmission between cats (the definitive host) and rodents (intermediate hosts). Rats are ubiquitous and *T. gondii* is asymptomatic in rats. Cats can excrete millions of environmentally resistant oocysts that can contaminate food and water, including marine waters. The authors here found that *T. gondii* can affect behaviour of rats. The authors chose wild-infected rats to prove that *T. gondii* affected their behaviour. Rats are nocturnal; infected rats were more active at night than uninfected rats. They are more likely to be preyed upon by cats.

Subsequently, Webster and associates proposed that infected rodents lost fear of cats and were attracted towards them; this proved true not only of domestic cats but also of wild felids (Webster, 2001). Subsequent studies by others showed that the neurotropic transmitter, dopamine, is concentrated in tissue cysts of *T. gondii* (reviewed in Dubey, 2022). This hypothesis concerning *T. gondii* affecting feline and rodent behaviour has been extended to human culture and entrepreneurship (Lafferty, 2006; Johnson et al., 2018).

4. **Flegr J, Zitková S, Kodym P, and Frynta D** (1996) Induction of changes in human behaviour by the parasitic protozoan *Toxoplasma gondii. Parasitology* **113**, 49–54, PMID: 8710414.

Approximately one-third of humanity has been exposed to *T. gondii*, mostly in latent form. The effects of chronic (latent) *T. gondii* infection in humans are undetermined. Flegr and associates have published many papers on this topic in the last 3 decades. This paper found a correlation between altered personality and seropositivity to *T. gondii*, including ego weakness and low intelligence, admitting the difficulty of establishing a causal path connecting latent chronic *T. gondii* infection with aspects of human behaviour or general well-being.

Based on many reports, by Flegr and his group and by others, *T. gondii* seropositivity has been linked to lower reaction time, tendency for accidents, suicides, neuroticism, memory loss and numerous mental disorders, and entrepreneurship behaviours (Dubey, 2022).

5. **Speer CA and Dubey JP** (1998) Ultrastructure of early stages of infection in mice fed *Toxoplasma gondii* oocysts. *Parasitology* **116**, 35–42. doi: 10.1017/S0031181097001959.

**Figure 1.**
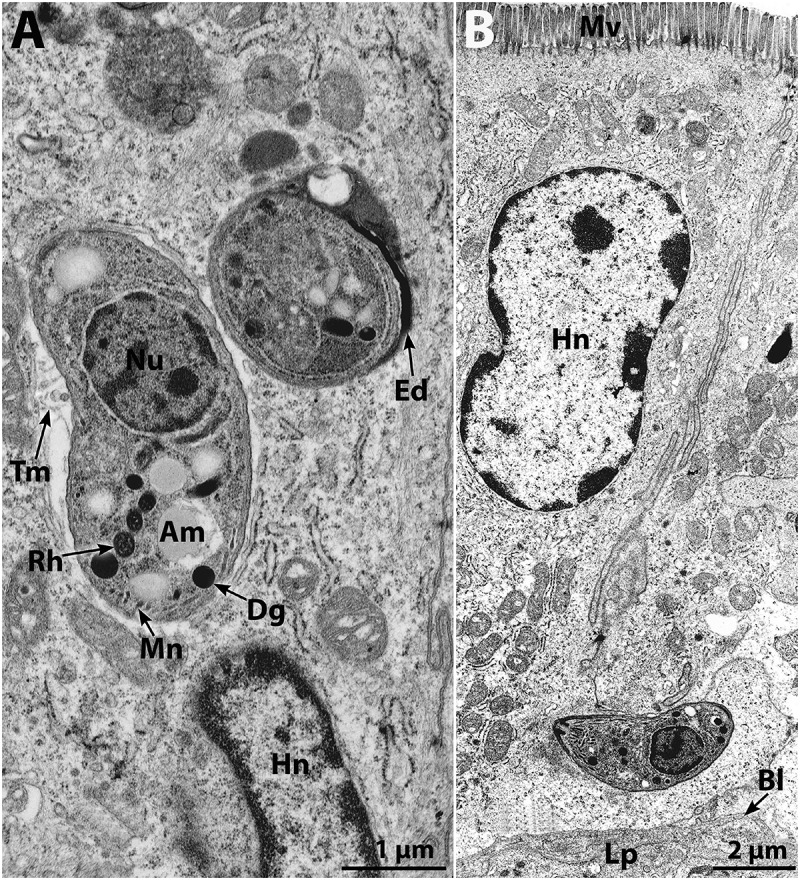
(A and B) Transmission electron , microscopy of sporozoites of the VEG strain of *Toxoplasma gondii* in mouse ileum at 2 h post-feeding of oocysts. (A) Two intracellular sporozoites located above the Hn of an ileal enterocyte; note the Ed in the parasitophorous vacuole surrounding the sporozoite cut in cross-section and the Tm in the other sporozoite. (B) Sporozoite located at the base of an ileal enterocyte. Abbreviations: Ed, electron-dense material; Tm, tubulovesicular membranous network; Am, amylopectin; Dg, dense granule; Mn, microneme; Nu, nucleus of sporozoite; Rh, rhoptry; Bl, basal lamina of intestinal epithelium; Hn, host cell nucleus; Mv, microvilli of enterocyte; Lp, lamina propria (from Speer and Dubey, 1998, *Parasitology* **116**, 35–42).

Before the discovery of the coccidian phase (oocyst) in the life cycle of *T. gondii* in 1970, all coccidia were presumed host specific and limited to a faecal oral cycle. By contrast, *T. gondii* has a very wide host range. Early on, it was assumed that the biology of *T. gondii* sporozoite would resemble that of other coccidian sporozoites. Limited studies with *Eimeria*, mainly using ligated intestinal loops inoculated with oocysts, indicated that excysted sporozoites of some *Eimeria* species penetrated intraepithelial lymphocytes (IELs) in intestinal epithelial and were transported to their final site of asexual development. Little was known of the mechanism of transport or of the early events of stage conversion from free-living sporozoite to the trophozoite form (reviewed in Dubey et al., 2020). This paper employed transmission electron microscopy to study the fate of sporozoites of *T. gondii* in the small intestines of mice at 2–48 h post-feeding (p.f.). Within 2 h, sporozoites passed through intestinal epithelial cells (enterocytes and goblet cells). Sporozoites penetrated enterocytes with their apical ends and were located within a parasitophorous vacuole, which contained exocytosed, electron dense material and well-developed tubulovesicular membranous networks. Sporozoites were not found in IELs. At 6 h p.f., sporozoites migrated to the lamina propria, where they infected all cell types except erythrocytes; none were then seen in the surface epithelium. At 12 h p.f., most sporozoites had divided into tachyzoites. At 24 h p.f., tachyzoites were confined to the lamina propria, but by 48 h p.f., infection had spread to surface epithelium, where tachyzoites infected enterocytes, goblet cells and IELs. This study showed, for the first time, that *T. gondii* sporozoite is very motile, penetrating enterocytes and reaching the base of enterocytes by 2 h p.f. (Figure 1). The conversion of sporozoite (free-living stage) to tachyzoite stage (parasitic form) occurred in the lamina propria, and the intestinal epithelium was parasitized by tachyzoites that originated in the lamina propria.

These observations provided new information on pathogenic mechanisms of acute toxoplasmosis in a natural setting. This was the first study that ultrastructurally documented *in vivo* early phases of stage conversion from sporozoite to tachyzoite or trophozoite of any coccidian sporozoite. Sporozoite-induced infections are more pathogenic, irrespective of the dose. Bioassay studies have since shown that *T. gondii* sporozoite can circulate in extraintestinal tissues (was isolated from orbital sinus blood of mice 4 h p.f.) but multiplies first in the lamina propria of mice (Dubey, 2022).

6. **Ferguson DJP, Jacobs D, Saman E, Dubremetz J-F, and Wright SE** (1999) In vivo expression and distribution of dense granule protein 7 (GRA7) in the exoenteric (tachyzoite, bradyzoite) and enteric (coccidian) form of *Toxoplasma gondii. Parasitology* **119**, 259–265, PMID: 10503251.

Dense granules (DGs) are important organelles of *T. gondii* and their contents are excreted into parasitophorous vacuole (PV). Several molecules located within DGs are released in PV and are designated as GRA 1–7 proteins. Previously, GRA 1–7 had been characterized in tachyzoites and bradyzoites. Here, the authors studied the *in vivo* expression and distribution of one DG protein. GRA7 was examined in both the exoenteric (tachyzoite and bradyzoite) and enteric (coccidian) forms of *T. gondii* by immunocytochemistry. There was strong staining of GRA7 in granules within all the infectious stages (tachyzoite, bradyzoite, merozoite and sporozoite). During tachyzoite development, GRA7 was secreted and was associated with the PV. In contrast, GRA 7 was demonstrated within the bradyzoites of more mature cysts; there appeared to be little or no staining of the tissue cyst wall or host cell. In the enteric forms in the cat intestine, strong labelling of the PV containing early asexual and sexual stages and staining of a few granules in the apical cytoplasm of the merozoite differentiates GRA7 localization from the other GRA proteins (GRA1–GRA6), which are absent from merozoites or enteric stages.

Subsequent research by others has shown that the GRA7 is important in inducing protective immunity (reviewed in Yang et al., 2016).

7. **Couzinet S, Dubremetz JF, Buzoni-Gatel D, Jeminet G, and Prensier G** (2000) *In vitro* activity of the polyether ionophorous antibiotic monensin against the cyst form of *Toxoplasma gondii. Parasitology* **121**, 359–365, PMID: 11072898.

Polyether ionophorous antibiotics are membrane active agents that collapse transmembrane ionic gradients necessary for cellular life. Some of these ionophores (e.g. the sodium ionophore monensin) were used in livestock production, particularly to prevent coccidiosis. This study examined the activity of the polyether ionophorous antibiotic monensin against the cyst form (bradyzoite) of *T. gondii*. The experiments were conducted *in vitro* using 2 methods: cysts produced either in mice or in cell culture were exposed to monensin *in vitro*, and their infectivity then assessed *in vivo* or *in vitro*. The data obtained from these 2 systems of evaluation showed that monensin inhibits the infectivity and the viability of the bradyzoites. Its activity was time and concentration dependent. The least effects were observed at very low drug concentrations. Immunofluorescence and electron microscopy analysis showed significant cytological alterations of the monensin-treated bradyzoites; they were swollen and had many cytoplasmic vacuoles. High concentrations induced parasite lysis.

Although many medicines are active against tachyzoites, monensin proved active against the chronic encysted form of *T. gondii*. Other authors have found prophylactic treatment of ewes with monensin to reduce their susceptibility to foetal mortality from toxoplasmosis (Buxton et al., 1988).

8. **Basso W, Edelhofer R, Zenker W, Möstl K, Kübber-Heiss A, and Prosnl H** (2005) Toxoplasmosis in Pallas’ cats (*Otocolobus manul*) raised in captivity. *Parasitology* **130**, 293–300, PMID: 15796012.

Pallas’ cats (*Felis manul*, syn. *Otocolobus manul*) are threatened, small felids that inhabit the high mountains of Tibet, western Siberia, Turkestan, western China and Mongolia. A global conservation effort seeks to breed them in captivity. Unlike humans, Pallas’ cats infected with *T. gondii* before pregnancy can repeatedly transmit *T. gondii* to their kittens. Additionally, they can excrete *T. gondii* oocysts. Between 1995 and 2000, many (>50%) of kittens born in North American captivity from mothers imported from Russia and Mongolia died within 4 months of birth. In most deceased kittens, toxoplasmosis was identified as the cause of death. Pallas’ cats held in captivity proved highly susceptible to toxoplasmosis and excreted many *T. gondii* oocysts. This study provided a detailed investigation of the problem in a zoo, documenting 58% mortality, between the 2nd and 14th week of life, in 24 kittens. Most deaths resulted from acute toxoplasmosis. The epidemiology of toxoplasmosis in Pallas’ cats was examined and a control strategy to protect the kittens from fatal toxoplasmosis was developed. One 12-week-old kitten, from a litter of 6 born in 2001, died of generalized toxoplasmosis. This kitten had excreted *T. gondii* oocysts bioassayed in mice. *Toxoplasma gondii* was isolated in tissue culture inoculated with tissues of these mice. Surviving kittens were immediately treated with clindamycin for 16 weeks; they acquired a natural infection and seroconverted but showed no clinical signs.

The preventive measures outlined here are used to prevent toxoplasmosis in Pallas’ cats in zoos. This has advanced conservation goals and has reduced human exposure to oocysts that would have otherwise been excreted by queens and their kittens.

9. **Parameswaran N, O’Handley RM, Grigg ME, Wayne A, and Thompson RCA** (2009) Vertical transmission of *Toxoplasma gondii* in Australian marsupials. *Parasitology*, **136**, 939–944, PMID: 19549348; PMCID: PMC4029946.

Vertical (transplacental or transmammary) transmission of *T. gondii* and its influence on the maintenance of *T. gondii* in natural populations have been a matter of debate. It had long been demonstrated, experimentally, that congenital infection with *T. gondii* in mice and guineapigs results when the dam is chronically infected. Until this study, however, little was known about the dynamics of vertical transmission of *T. gondii* in Australian marsupials. In this study, polymerase chain reaction and immunohistochemistry were used to detect *T. gondii* in chronically infected marsupial dams and their offspring. *Toxoplasma gondii* was detected in the unfurred pouch young of 2 out of 10 chronically infected western grey kangaroos (*Macropus fuliginosus*) and in the unfurred pouch young of a brush-tailed bettong (*Bettongia penicillata*). Results of the study suggest that vertical transmission of *T. gondii* can occur in chronically infected Australian marsupials.

Information on the occurrence of vertical transmission in chronically infected marsupials benefited captive breeding programmes of Australian marsupials by ensuring only *T. gondii*-free animals are bred, thereby improving animal health and assisting animal conservation and management.

10. **Innes EA, Bartley PM, Buxton D, and Katzer F** (2009) Ovine toxoplasmosis. *Parasitology* **136**, 1887–1894, PMID: 19995468.

The most important clinical consequence of *T. gondii* in livestock concerns its role as an abortifacient in sheep. Millions of lambs are lost yearly because of toxoplasmosis. Abortions due to toxoplasmosis were first recognized in the 1950s in Australia and New Zealand by Hartley et al. (1954). These findings were confirmed in England by Beverley and Watson (1961) in the 1960s (reviewed in Dubey and Beattie, 1988). Subsequently, research performed at the Moredun Research Institute, Edinburgh, Scotland contributed immensely to the overall understanding of ovine toxoplasmosis epidemiology and control. This paper summarized ovine toxoplasmosis, particularly the role played by *T. gondii* oocysts. Ingestion of just a few oocysts can cause abortion, usually once in a ewe’s lifetime; sheep that have aborted due to toxoplasmosis develop protective immunity for subsequent abortions. The group at the Moredun Research Institute was instrumental in the development of the first commercial vaccine, Toxovac. Inoculating sheep with tachyzoites of the vaccine strain (S48) before mating afforded protection against *T. gondii* induced abortion. This vaccine does not prevent infection of the foetus but does reduce damage to the foetus. This protection persists for at least 18 months.

This is an authoritative review of ovine toxoplasmosis. It questions claims by another group of researchers in England that suggested repeated abortion and congenital infections in sheep.

11. **Su C, Shwab EK, Zhou P, Zhu XQ, and Dubey JP** (2010) Moving towards an integrated approach to molecular detection and identification of *Toxoplasma gondii. Parasitology*. **137**, 1–11, PMID: 19765337.

Until the development of molecular methods for genotyping in the 1990s, *T. gondii* were usually grouped as virulent or non-virulent based on pathogenicity in mice (Dubey and Beattie, 1988). The development of multi-locus enzyme electrophoresis, polymerase chain reaction (PCR)-based restriction fragment length polymorphism (RFLP) and microsatellite analysis allowed grouping of *T. gondii* into different genetic lineages. However, conflict characterized results obtained by these different techniques. This paper developed a multiplex PCR to standardize the RFLP using 10 genetic markers (SAG1, SAG2, SAG3, BTUB, GRA6, c22-8, c29-2, L358, PK1 and Apico), enabling identification of reference *T. gondii* strains.

This technique provided a simple and cost-effective typing method that has a reasonable resolution for *T. gondii* genotyping. It has been used widely for studying molecular epidemiology of *T. gondii* and made significant contribution to the understanding of global genetic diversity, population structure and transmission of this parasite (Dubey, 2022).

12. **Dubey JP, Ferreira LR, Martins J, and McLeod R** (2012) Oral oocyst-induced mouse model of toxoplasmosis: Effect of infection with *Toxoplasma gondii* strains of different genotypes, dose, and mouse strains (transgenic, out-bred, in-bred) on pathogenesis and mortality. *Parasitology* **139**, 1–13, PMID: 22078010; PMCID: PMC3683600.

Mice play an indisputably important role in studying toxoplasmosis, including tests of the protective efficacy of vaccines, because they are susceptible, easily managed in the laboratory, and well characterized with readily available immunological reagents. Caution is warranted in overinterpreting a given strain’s virulence in laboratory mice: such sequalae vary according to mouse genetics, route of inoculum, dose and the stage of parasite administered. Oocysts are generally more pathogenic than tissue cysts. Moreover, outcomes vary across host species, limiting the predictive power of mice for understanding strain-specific risks to human health.

This paper achieved detailed insights into the interaction of host and parasite genotype to advance the evaluation of vaccine candidates to prevent human disease, studying pathogenesis in mice of different strains, including Human Leukocyte Antigen (HLA) transgenic mice, infected with varied doses of multiple parasite strains. In decreasing order of oocyst infectivity and pathogenicity, they discovered: C57BL/6 background interferon gamma gene knock out, HLA-A*1101, HLA-A*0201, HLA-B*0702, Swiss Webster, C57/black and BALB/c. Mice fed as few as 1 oocyst of Type I, or of several atypical strains, died of acute toxoplasmosis within 21 days post-infection. Some Type II and III strains were less virulent. The model developed herein established an extremely useful scheme for testing vaccines by demonstrating the possibility of accurately quantifying a challenge inoculum, testing responses of genetically variable mice to a given preparation of parasite oocysts (which are stable up to a year) and achieving highly reproducible responses to infection.

This paper was of special personal significance to me. Ignoring medical advice, I stayed late at the office so that I could submit the paper before my cardiac bypass surgery scheduled next morning. I also telephoned co-author Dr Rima Mcleod to ensure that she would ensure manuscript’s publication in case I did not wake from surgery.

13. **Dubey JP, Lago EG, Gennari SM, Su C, and Jones JL** (2012) Toxoplasmosis in humans and animals in Brazil: High prevalence, high burden of disease, and epidemiology. *Parasitology*. **139**, 1375–1424, PMID: 22776427.

Until the 20th century, little attention was paid to toxoplasmosis in Brazil or other parts of South America. Reports by Dubey et al. (2002) and Lehmann et al. (2006) discovered that isolates of *T. gondii* from asymptomatic feral chickens from Brazil differed from North American isolates in genotype and phenotype; *T. gondii* isolates were lethal for outbred laboratory mice and the strains were non-clonal. Infections by *T. gondii* are widely prevalent in humans and animals in Brazil. The high clinical burden of human toxoplasmosis in humans lacked requisite international attention because early papers documenting this were published in Portuguese and were often not available to scientists in English-speaking countries. This 50-page paper reviewed prevalence, clinical spectrum, molecular epidemiology and control of *T. gondii* in humans and animals in Brazil. A very high rate of *T. gondii* infection in humans was documented. Up to 50% of elementary school children and 50–80% of women of child-bearing age had antibodies to *T. gondii*. The risks for uninfected women to acquire toxoplasmosis during pregnancy and foetal transmission are high because the environment is highly contaminated with oocysts. The burden of toxoplasmosis in congenitally infected children is also very high. Most of congenitally infected children are likely to develop symptoms or signs of clinical toxoplasmosis. Among the congenitally infected children whose clinical data are described in this review, several died soon after birth; 35% had neurological disease including hydrocephalus, microcephaly and mental retardation; 80% had ocular lesions; and in one report, 40% of children had hearing loss.

The severity of clinical toxoplasmosis in Brazilian children has been speculated to be associated with the genetic characteristics of *T. gondii* isolates prevailing in animals and humans in Brazil. However, in my own view, it could be related to the dose and the source of infection; the environment in Brazil is highly contaminated with oocysts. Compared with disease in humans, little is known for clinical disease in animals, particularly livestock. This review contained *T. gondii* prevalence data for all domestic species of livestock.

14. **Shwab EK, Zhu XQ, Majumdar D, Pena HFJ, Gennari SM, Dubey JP, and Su C** (2014) Geographical patterns of *Toxoplasma gondii* genetic diversity revealed by multilocus PCR-RFLP genotyping. *Parasitology* **141**, 453–461, PMID: 24477076.

Until the 20th century, *T. gondii* was considered clonal with little genetic variability among isolates. This study assembled genetic data from 1457 *T. gondii* isolates worldwide and grouped them into 189 genotypes. Of these, just a few dominate in temperate regions of the northern hemisphere, which is in stark contrast to the southern hemisphere, especially in the tropics, where hundreds of genotypes coexist and where none dominate. PCR-RFLP genotypes #1 (Type II clonal), #2 (Type III), #3 (Type II variant) and #10 (Type I) occur globally. Genotypes #2 and #3 dominate in Africa, genotypes #9 (Chinese 1) and #10 are prevalent in Asia, genotypes #1, #2 and #3 are prevalent in Europe and genotypes #1, #2, #3, #4 and #5 dominate in North America (#4 and #5 are collectively known as Type 12). In Central and South America, no genotype dominates even though a few have relatively high frequencies. Statistical analysis indicated significant differences among populations in Africa, Asia, Europe, North America and Central and South America, but Europe and North America (the regions heretofore most studied, and therefore affording a biased view of global patterns) exhibited notable similarity. Collectively, the results revealed distinct population structures and geographical patterns of diversity in *T. gondii.*

This was the first worldwide evaluation of genetic diversity of *T. gondii*. This is a milestone publication and comprehensive evaluation of genetic diversity of *T. gondii* worldwide based on the PCR-RFLP methodology published in reference 11 mentioned earlier. It significantly advanced our understanding of *T. gondii* population structure since the earlier milestone publication of Howe and Sibley (1995) that established the designation of Type I, II and III linages.

15. **Dubey JP, Laurin E, and Kwok OCH** (2016) Validation of the modified agglutination test for detection of *Toxoplasma gondii* in free-range chickens by using cat and mouse bioassay. *Parasitology* **143**, 314–319. doi: 10.1017/S0031182015001316.

The Sabin–Feldman dye test is the most specific and sensitive test for the detection of antibodies to *T. gondii* in human sera and many species of animals. However, the dye test does not work for the detection of antibodies in chickens and cattle. The use of live virulent *T. gondii* tachyzoites and the need for accessory factor present only in human sera required for the dye test prevented it from commercialization, and the test was performed in only a few laboratories worldwide. The development of an agglutination test, now called the modified agglutination test (MAT), in the 1980s (Desmonts and Remington, 1980; Dubey and Desmonts, 1987) facilitated its widespread use and commercialization. However, there were only a few limited data concerning sensitivity and specificity of MAT (or for any serological test for *T. gondii*) because verification required isolation of viable *T. gondii* from naturally infected animals. In this paper, the authors evaluated the diagnostic accuracy of the MAT and bioassay in free-range/backyard (FR) chickens (*Gallus domesticus*) from 2066 chickens from 19 countries. The frequency of isolation of *T. gondii* increased for MAT titres between 1:5 and 1:160. Twenty-three cats fed pooled hearts from a total of 802 FR seronegative (MAT < 1:5) chickens from several countries did not excrete oocysts, indicating a high negative predictive value of MAT because FR chickens would have been exposed to many microbes; cats are the most sensitive indicators of *T. gondii* infection in tissues and can excrete millions of oocysts after ingesting even a few bradyzoites. Results of the present study supported the validity of MAT for the detection of *T. gondii* infection in chickens. The results indicated that the detection of even low antibody titres is epidemiologically important.

The MAT has been used widely for serological diagnosis of *T. gondii* infections in humans and animals. It has been modified to detect IgM antibodies (Dubey, 2022).

16. **Schott KC, Krusor C, Tinker MT, Moore J, Conrad PA, and Shapiro K** (2016) Concentration and retention of *Toxoplasma gondii* surrogates from seawater by red abalone (*Haliotis rufescens*). *Parasitology* **143**, 1703–1712, PMID: 27573192.

*Toxoplasma gondii* can cause mortality in many species of endotherms, including marine mammals. Serological and autopsy data indicate that the prevalence of *T. gondii* in sea otters in California, USA, is very high; and higher *T. gondii* seroprevalence is associated with higher human density, suggesting an association with felids. How marine mammals become infected with *T. gondii* is enigmatic because they feed mainly on ectothermic aquatic life. One hypothesis is that oocysts from land runoff water concentrate in bivalves, which act as paratenic hosts. Sea otters consume approximately one-third of their body weight daily. This study used red abalone (*Haliotis rufescens*) and 10.4 µm microsphere surrogates for *T. gondii* oocysts to determine whether abalone can acquire and retain *T. gondii.* Abalone were exposed to *T. gondii* surrogate microspheres for 24 h, and faecal samples were examined for 2 weeks following exposure. Surrogates proved 2–3 orders of magnitude more concentrated in abalone faeces than in the spiked seawater, and excretion of surrogates continued for 14 days post-exposure.

These results indicate that, like snails, abalone can serve as vectors of *T. gondii* as paratenic hosts. These results may be applicable to related pathogens, such as *Sarcocystis neurona*, which causes mortality in sea otters when they ingest oocysts excreted from the Virginia opossum, *Didelphis viginianus*.

17. **Dubey JP, Cerquiera-Cézar CK, Murata FHA, Verma SK, Kwok OCH, Pedersen K, Rosenthal BM, and Su C** (2020) Genotyping of viable *Toxoplasma gondii* from the first national survey of feral swine revealed evidence for sylvatic transmission cycle, and presence of highly virulent parasite genotypes. *Parasitology* **147**, 295–302, PMID: 31739817; PMCID: PMC10317635.

Feral swine (*Sus scrofa*) populations in the USA are estimated to exceed 5 million, and their geographic range continues to expand. Feral swine pose a threat to non-biosecure domestic pig facilities by serving as reservoirs for pathogens, which may be transmitted to domestic pigs. The presence of *T. gondii* in feral swine is considered a good indicator of contamination in the environment because they are omnivores with a generalist diet and can become infected by ingesting oocysts while rooting and eating tissues of infected animals. Transmission of *T. gondii* has been documented in free-ranging domestic pigs through cannibalism. The objective of the present investigation was to isolate and characterize *T. gondii* from feral swine across the USA. This study reported the first national survey of viable *T. gondii* in feral swine in the USA. Serological surveys were paired with parasite isolation and bioassay to evaluate the prevalence and genetic diversity of these parasites. From 2012 to 2017, sera and tissues from 1517 feral swine across the USA were collected for the isolation of viable *T. gondii*. Serum samples were initially screened for antibodies to *T. gondii*, and then the tissues of seropositive feral swine were bioassayed in mice. Antibodies were detected in 27.7% of feral swine tested by the MAT (1:25 or higher). Antibody-positive rates increased significantly with age, with 10.1% of juveniles, 16.0% of sub-adults and 38.4% of adults testing seropositive, indicating post-natal transmission of *T. gondii*. Myocardium (50 g) from 232 seropositive feral swine was digested in pepsin and bioassayed in mice. Viable *T. gondii* was isolated from 78 feral swine from 21 states. Twelve of the 78 isolates were pathogenic to outbred Swiss Webster mice and 76 of the 78 isolates could be propagated further in cell culture and were genotyped using 11 PCR-RFLP markers. Genotyping revealed 15 ToxoDB genotypes. Genotype #5 was the most frequently isolated, accounting for 57% (43/76) of the isolates. One genotype (genotype #289) was highly virulent to mice and originated from feral swine collected in Louisiana on the same day at the same location. *Toxoplasma gondii* isolates from Hawaii were genetically different than the isolates from Continental USA.

The results indicated that the urban environment is highly contaminated with *T. gondii* and mouse virulent *T. gondii* strains exist in asymptomatic feral swine.

18. **Dubey JP, Cerqueira-Cézar CK, Murata FHA, Verma SK, Kwok OCH, Pedersen K, Rosenthal BM, and Su C** (2020) White-tailed deer (*Odocoileus virginianus*) are a reservoir of a diversity of *Toxoplasma gondii* strains in the USA and pose a risk to consumers of undercooked venison. *Parasitology* **147**, 775–781, PMID: 32178743; PMCID: PMC1031854.

Humans become infected with *T. gondii* postnatally by ingesting food and water contaminated with the environmentally resistant oocysts excreted in felid faeces and by eating undercooked infected meat. The sources of infections (whether oocysts or infected meat) are difficult to determine. Estimating their relative importance relied on reported food habits and occupational exposure risk. A serological test that distinguishes oocyst- versus meat-acquired infections (Hill et al., 2011) indicated that the transmission by oocysts is more prevalent than previously appreciated. Assessment of faecal (oocyst) contamination is very difficult, requiring testing of vast amounts of soil and water with methods that lack sensitivity. Deer, especially the White-tailed deer (WTD, *Odocoileus virginianus*), are widely prevalent in the USA, and they are strictly herbivorous. Thus, postnatal infection in deer derives exclusively from oocyst ingestion. This national survey of WTD combined serology with parasite isolation to evaluate the prevalence and genetic diversity of *T. gondii* in this game species. From October 2012 to March 2019, serum and tissues were collected from 914 WTD across the USA. Serum samples were screened for antibodies to *T. gondii*, and then the tissues of seropositive WTD were bioassayed in mice. Antibodies were detected in 329 (36%) of 914 WTD tested by the modified agglutination test (positive reaction at 1:25 or higher). Viable *T. gondii* was isolated from the hearts of 36 WTD from 11 states. Three of the 36 isolates were pathogenic, but not highly virulent, to outbred Swiss Webster mice; all 36 isolates were propagated further in cell culture and were genotyped. DNA extracted from cell culture-derived tachyzoites was characterized by PCR-RFLP using the genetic markers SAG1, SAG2, SAG3, BTUB, GRA6, c22-8, c29-2, L358, PK1 and Apico. Genotyping revealed 7 ToxoDB PCR-RFLP genotypes, including 24 isolates for genotype #5 (haplogroup 12), 4 isolates for genotype #2 (type III, haplogroup 3), 3 isolates for genotype #1 (type II, haplogroup 2), 2 isolates for genotype #3 (type II, haplogroup 2) and 1 isolate each for genotypes #39, #221 and #224. Genotype #5 was the most frequently isolated, accounting for 66.6% (24 of 36) of the isolates. Combining the 36 isolates from this study with previously reported 69 isolates from WTD, 15 genotypes have been identified from this herbivorous host. Among these, 50.4% (53/105) isolates belong to genotype #5. The results indicated moderate genetic diversity of *T. gondii* in WTD.

The results indicated that the urban environment in the USA is highly contaminated with oocysts and deer serve as good indicators of infections acquired from water and soil.

19. **Dubey JP, Pena HFJ, Cerqueira-Cézar CK, Murata FHA, Kwok OCH, Yang YR, Gennari SM, and Su C** (2020) Epidemiologic significance of *Toxoplasma gondii* infections in chickens (*Gallus domesticus*): the past decade. *Parasitology* **147**, 1263–1289, PMID: 32660653; PMCID: PMC10317745.

**Figure 2.**
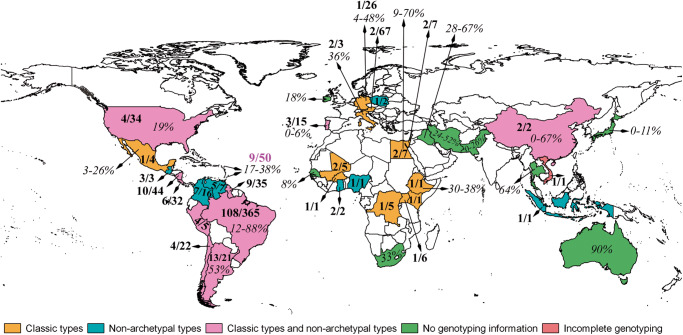
Worldwide distribution of *Toxoplasma gondii* infections in chickens. Numbers in bold are the number of *T. gondii* genotypes/number of viable isolates. Seroprevalences are given as % (from Dubey et al., 2020, *Parasitology* **147**, 1263–1289).

Until 2000, available data supported low genetic diversity and clonal propagation of *T. gondii*. Interest in genetic diversity of *T. gondii* was spurred after some isolates proved more virulent than others and certain genotypes were associated with clinical human toxoplasmosis. This study was the first to compare virulence of different *T. gondii* isolates using a standardized protocol. Beginning in 2000, a collaborative research project was initiated at the U.S. Department of Agriculture facility in Beltsville, MD, USA; the project was terminated in 2019. The main objective was to study the genetic diversity of *T. gondii* using DNA derived from live parasites. Our initial focus was South America because, until then, little was known about the genetic diversity of *T. gondii* in this part of the world. Tissues from chickens were bioassayed in outbred Swiss Webster mice and in cats. Thus, the biology of each isolate was assessed using uniform protocols. The ready availability and low cost of chickens, as well as their feeding behaviour (pecking at the ground, drinking surface water), favoured their use. Moreover, no restrictions barred importation of chicken tissues at that time. (A decade later, such restrictions were imposed in hopes of preventing H5N1 influenza importation.) Great success resulted from collaborations with Brazilian scientists in several institutions, orchestrated by Prof. Solange Gennari from São Paulo. This study succeeded in isolating viable *T. gondii* from most regions of Brazil (Figure 2). This labour-intensive and costly research entailed a door-to-door survey of houses with backyard chickens. To ensure sampling independence, no more than 10 chickens were sampled from any given farm/house, and these farms were separated from one another by approximately 1 km. Chickens purchased from individual households were housed at a local facility and euthanized just prior to shipping via overnight courier service to Beltsville for bioassay. Success in Brazil impelled the study’s extension to 19 other countries. At Beltsville, each tissue sample was bioassayed in 5 mice to assess virulence. A naming system designated each *T. gondii* isolate: Tg (for *T. gondii*), Ck (for chicken) and the country code (e.g. Br for Brazil). Genotyping was performed using PCR-RFLP, and the results summarized all data using 10 PCR-RFLP. This study assessed prevalence, infection persistence, clinical disease, epidemiology and genetic diversity of *T. gondii* strains isolated from chickens, worldwide, over a decade (Figure 2). Data on phenotypic and molecular characteristics of 794 viable *T. gondii* strains from chickens were discussed, including new data on *T. gondii* isolates from chickens in Brazil.

Recently, these studies were extended to a survey of 401 feral chickens in Amazonia, including Brazil, Peru, Bolivia and Guyana; 273 (68.1%) of chickens were seropositive and viable *T. gondii* was isolated from the hearts of 116 seropositive chickens (Gennari et al., 2024). Genetic typing revealed 89 genotypes, including 20 new genotypes.

20. **Dubey JP, Murata FHA, Cerqueira-Cézar CK, Kwok OCH, and Villena I** (2021) Congenital toxoplasmosis in humans: An update of worldwide rate of congenital infections. *Parasitology* **148**, 1406–1416, PMID: 34254575; PMCID: PMC11010219.

Clinical congenital toxoplasmosis is the most important aspect of *T. gondii* infection. The morbidity due to congenital toxoplasmosis in humans is very high. Most of these infected children are likely to develop symptoms of clinical toxoplasmosis. Foetal sequelae, especially from infections acquired by mothers during pregnancy, can be devastating; some countries direct enormous effort to prevent these consequences. This study reviewed global rates of congenital human toxoplasmosis. Although several countries have surveillance programmes, most information on the rate of congenital transmission is from France and Brazil. Compulsory national screening in France detects and treats women with recently acquired *T. gondii* infection; hence, the rate of congenital transmission and the severity of disease in children are declining. The risk of congenital infection is the lowest when the mother becomes infected during the first trimester (10–15%); that risk is the highest when the mother acquires infection during the third trimester. If maternal infection occurs early in pregnancy, it results in fewer infected babies, but they are more severely affected than the greater number of infected babies born when infection is acquired later in pregnancy. The highest risk to the foetus occurs when infection is acquired between the 10th and 24th week of gestation. This study tabulated 30 years of studies from France. Infections by this parasite are widely prevalent in Brazil. The severity of clinical toxoplasmosis in Brazilian children is very high, perhaps exacerbated by heritable characteristics of regionally endemic strains. This study also tabulated worldwide data from prenatal or postnatal screening programmes. Unfortunately, little information was available from China, India or other countries in Asia.

This paper provides quantitative data on the rate of congenital toxoplasmosis worldwide based on prenatal and postnatal screening of women.
